# Norepinephrine mediates heart block during severe hypoglycemia in male rats

**DOI:** 10.14814/phy2.70010

**Published:** 2024-08-23

**Authors:** Emily H. Nuibe, Matthew E. Chambers, Candace M. Reno‐Bernstein

**Affiliations:** ^1^ Division of Endocrinology, Metabolism, and Diabetes University of Utah School of Medicine Salt Lake City Utah USA

**Keywords:** animal model, arrhythmias, diabetes, myocardial infarction, norepinephrine, severe hypoglycemia

## Abstract

Hypoglycemia is common in people with type 1 diabetes. Sometimes, severe hypoglycemia can be fatal. The underlying mechanisms by which severe hypoglycemia can lead to death are unclear. The sympathetic nervous system is thought to be proarrhythmic. We hypothesized that norepinephrine is the main mediator of severe hypoglycemia‐induced fatal cardiac arrhythmias. To test this hypothesis, adult, non‐diabetic Sprague–Dawley rats were subjected to hyperinsulinemic‐severe hypoglycemic clamps (3 h, 10–15 mg/dL) during two different experiments: (1) intracerebroventricular (ICV) norepinephrine (*n* = 26) or artificial cerebrospinal fluid (aCSF) (*n* = 20) infusion or (2) blockade of norepinephrine release by intraperitoneal reserpine (*n* = 20) or control (*n* = 29). In experiment 1, brain norepinephrine infusion during severe hypoglycemia led to a 2.5‐fold increase in third‐degree heart block and a 24% incidence of ST elevation compared to no ST elevation in aCSF controls. In experiment 2, reserpine successfully reduced plasma and cardiac norepinephrine levels. During severe hypoglycemia, reserpine completely prevented second and third‐degree heart block and T wave increases, a marker of myocardial infarction, compared to controls. In conclusion, norepinephrine increases while reserpine, used to reduce norepinephrine nerve terminal release, reduces heart block and markers of myocardial infarction during severe hypoglycemia.

## 1 INTRODUCTION

Iatrogenic hypoglycemia during the treatment of diabetes remains a limitation in disease management. Specifically, in type 1 diabetes, severe hypoglycemia has an annual incidence of 3.3%–13.5% (Pettus et al., 2019). Hypoglycemia can lead to seizures, brain damage, and coma (Abdelmalik et al., 2007). If hypoglycemia is severe, it can also be lethal, especially in young people with type 1 diabetes, accounting for up to 10% of deaths among this population (Skrivarhaug et al., 2006). This phenomenon has been termed the ‘dead in bed’ syndrome, where people with type 1 diabetes are found dead in an undisturbed bed with no clear cause of death (Secrest et al., 2011). There are speculations that hypoglycemia is the direct cause of death in this syndrome (Tanenberg et al., 2010), but how hypoglycemia leads to death has been unclear.

Both clinical and animal studies have revealed that cardiac alterations and arrhythmias occur during hypoglycemia and may be the underlying cause of the ‘dead in bed’ syndrome (Reno et al., 2013; Robinson et al., 2003). During hypoglycemia, the brain senses low glucose and activates the sympathetic nervous system leading to epinephrine and norepinephrine secretion to raise blood glucose (Belfort‐DeAguiar et al., 2018). Although this response is necessary, it may also be contributing to cardiac arrhythmias as the sympathetic nervous system is known to be proarrhythmic. We have previously shown in a rat model that severe hypoglycemia‐induced fatal cardiac arrhythmias were prevented with beta‐adrenergic receptor blockade (Reno et al., 2013). However, a follow‐up paper showed that removal of the adrenal medulla to reduce epinephrine levels had no effect on severe hypoglycemia‐induced fatal cardiac arrhythmias (Reno et al., 2019). Together, these studies indicate that some other aspect of the sympathetic nervous system other than epinephrine may play an important role in cardiac arrhythmias during hypoglycemia.

Besides epinephrine, the sympathetic nervous system response also includes norepinephrine release from nerve terminals in various tissues (Atzori et al., 2016; Gordan et al., 2015). Since norepinephrine can also act on beta‐adrenergic receptors, it was hypothesized that norepinephrine is the main mediator of severe hypoglycemia‐induced fatal arrhythmias. To test this hypothesis, two studies were performed in rats during severe hypoglycemia: (1) brain norepinephrine infusion, and (2) peripheral norepinephrine nerve terminal blockade.

## 2 MATERIALS AND METHODS

### 2.1 Animals

Adult, male, Sprague–Dawley rats (8–12 weeks old, ~350 g) were purchased from Charles River Lab and were individually housed at room temperature in an animal facility in 12:12 h light: dark cycles and received chow (LabDiets, cat. # 5001) ad libitum. Rats were single‐housed in wood chip bedding with enrichment of a house and wood block to chew on. Rats were randomized 1 week after arrival at the facility for experimentation group.

Ethics Statement: All studies were done in accordance with the University of Utah School of Medicine and Institutional Animal Care and Use Committee (IACUC) and approval was sought from IACUC prior to the studies being done.

### 2.2 Vessel cannulation and ECG Lead placement

Rats underwent surgery for carotid artery and jugular vein cannulation and electrocardiogram (ECG) placement ~1 week prior to the hypoglycemic clamp (described below) (Reno et al., 2013). The ECG leads were placed with one wire in the right supraclavicular fossa and another exterior to the lower left rib cage and sutured to underlying muscle. A reference lead was placed subcutaneously.

### 2.3 Brain cannulation surgery

For experiment 1, immediately after vessel cannulation, rats underwent surgery for placement of an intracerebroventricular (ICV) cannula (internal cannula 31 g, outer guide cannula 24 g, 9.5 mm depth; Plastics One, Roanoke, VA). Guide cannulas were placed on the skull (−2.88 mm posterior to bregma).

### 2.4 Hyperinsulinemic/severe hypoglycemic clamp

One week after surgeries, overnight fasted, awake, unrestrained rats were subjected to a 3 h severe hypoglycemic clamp (10–15 mg/dL). Insulin (0.2 U/kg/min, Humulin R, Lily Research Labs, Indianapolis, Indiana, NDC 0002‐8215‐17) and dextrose (50%) were co‐infused intravenously to lower and maintain blood glucose. An electrocardiogram (ECG) (PowerLab 26 T; LabChart; ADInstruments, Colorado Springs, CO) was recorded throughout the clamp. Blood glucose was sampled via the carotid artery cannula and measured with a glucometer (Ascensia Contour BG monitors; Bayer Healthcare, Mishawaka, IN). ELISAs were performed for plasma and cardiac epinephrine and norepinephrine (Abnova, Taipei City, Taiwan, cat. # KA1877). Blood samples taken for epinephrine and norepinephrine were preserved with an EGTA‐Glutathione solution. After the clamp, hearts were excised and frozen immediately. Hearts were ground with EGTA‐Glutathione solution for preservation and placed in a small tube and kept cool. The beadbug homogenizer was used to quickly homogenize the tissue. Samples were frozen at −80°C until used in the assays. Cardiac arrhythmias were manually counted. QTc (Bazett's formula) and heart rate were measured and analyzed every 15 min. Respiration and seizure‐like activity were manually observed.

### 2.5 Experiment 1: Brain norepinephrine infusion

In one set of rats, the dose of ICV norepinephrine (Nore, 4 nM, *n* = 5; Tocris Biosciences, Bristol, United Kingdom, cat. # 5169) was tested compared to artificial cerebrospinal fluid (aCSF; *n* = 3; Harvard Apparatus, Holliston, Massachusetts, cat. # 59‐7316). On the day of the experiment, norepinephrine was dissolved in aCSF and 3 mM sodium metabisulfite to maintain stability. aCSF and norepinephrine were infused continuously for 3 h, and arterial blood glucose was measured to ensure a rise with ICV norepinephrine. During the severe hypoglycemic clamp, aCSF (*n* = 20) or norepinephrine (4 nM; *n* = 26) were continuously infused ICV throughout the duration of the clamp.

### 2.6 Experiment 2: Blockade of norepinephrine at nerve terminals

On the day of the clamp, rats received an intraperitoneal injection of either control (*n* = 29, DMSO/saline) or reserpine (reduces norepinephrine; 4 mg/kg; *n* = 20, Tocris Biosciences, Bristol, United Kingdom, cat. # 2742) ~1 h prior to the start of the clamp.

### 2.7 Statistics

All data are represented as mean ± SD. Fisher exact test with Freeman–Halton extension was used for analysis of mortality and incidence of arrhythmias. Student's *t*‐test (GraphPadPrism) was used for all other analyses. Significance was determined at *p* < 0.05.

## 3 RESULTS

### 3.1 Experiment 1: Brain norepinephrine infusion

**FIGURE 1:**
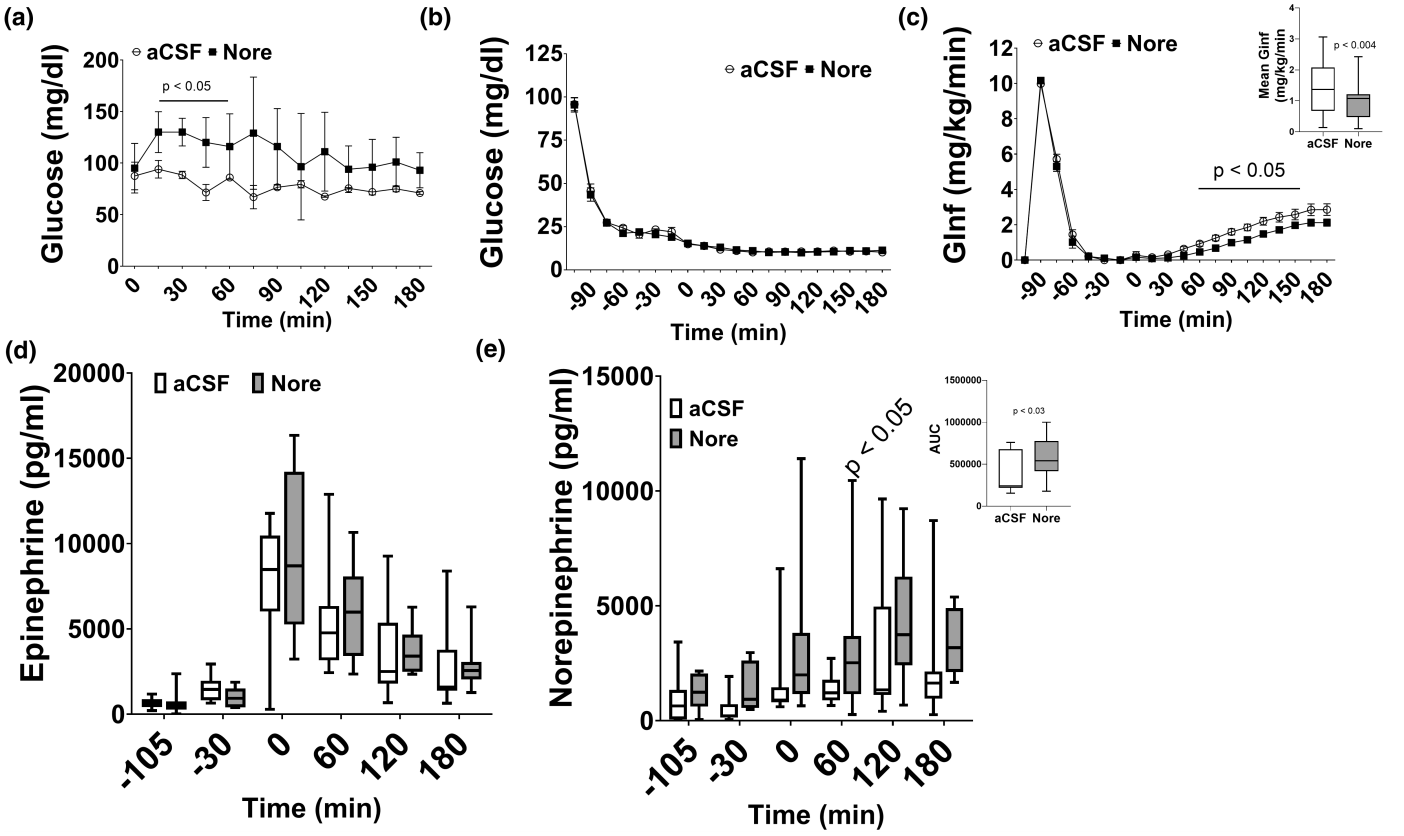
Experiment 1: Brain norepinephrine infusion clamp data. (a) Glucose levels during the dose response experiment showing glucose levels rise in response to norepinephrine infusion (black squares, *p* < 0.05 at indicated timepoints) compared to aCSF (white circles). (b) Glucose levels were evenly matched between aCSF (white circles) and Nore (black squares). (c) The glucose infusion rate required to maintain severe hypoglycemia was decreased at several timepoints in Nore (*p* < 0.05). Insert: The mean glucose infusion rate during severe hypoglycemia was decreased in Nore (gray bar; *p* < 0.004) compared to aCSF (white bar). (d) Plasma epinephrine peaked at the start of severe hypoglycemia (time 0) and decreased over the remaining 3 h. There were no differences between the groups. (e) Plasma norepinephrine was increased in Nore at 60 min into severe hypoglycemia compared to aCSF (*p* < 0.05). Insert: The area under the curve (AUC) was significantly increased in Nore compared to a CSF (*p* < 0.03). Data are represented as box and whisker plots with median and interquaritle range and min to max values. *N* = 20–26/group.

When ICV norepinephrine was infused in the absence of hypoglycemia, the blood glucose levels rose 1.38–1.92‐fold compared to aCSF controls (*p* < 0.05; Figure 1a) indicating an appropriate dose. During the severe hypoglycemic clamp, glucose levels were evenly matched between aCSF (11.4 ± 1.28 mg/dL) and Nore (11.8 ± 1.40 mg/dL) groups (Figure 1b). Brain norepinephrine infusion decreased the mean glucose infusion rate during severe hypoglycemia (0.79 ± 0.48; *p* < 0.004) compared to aCSF (1.4 ± 0.83; Figure 1c). Plasma epinephrine was not different between the two groups, but plasma norepinephrine was increased in norepinephrine (AUC: 592435 ± 254,770; *p* < 0.03) compared to aCSF (AUC: 361364 ± 240,021; Figure 1d,e) during severe hypoglycemia.

**FIGURE 2:**
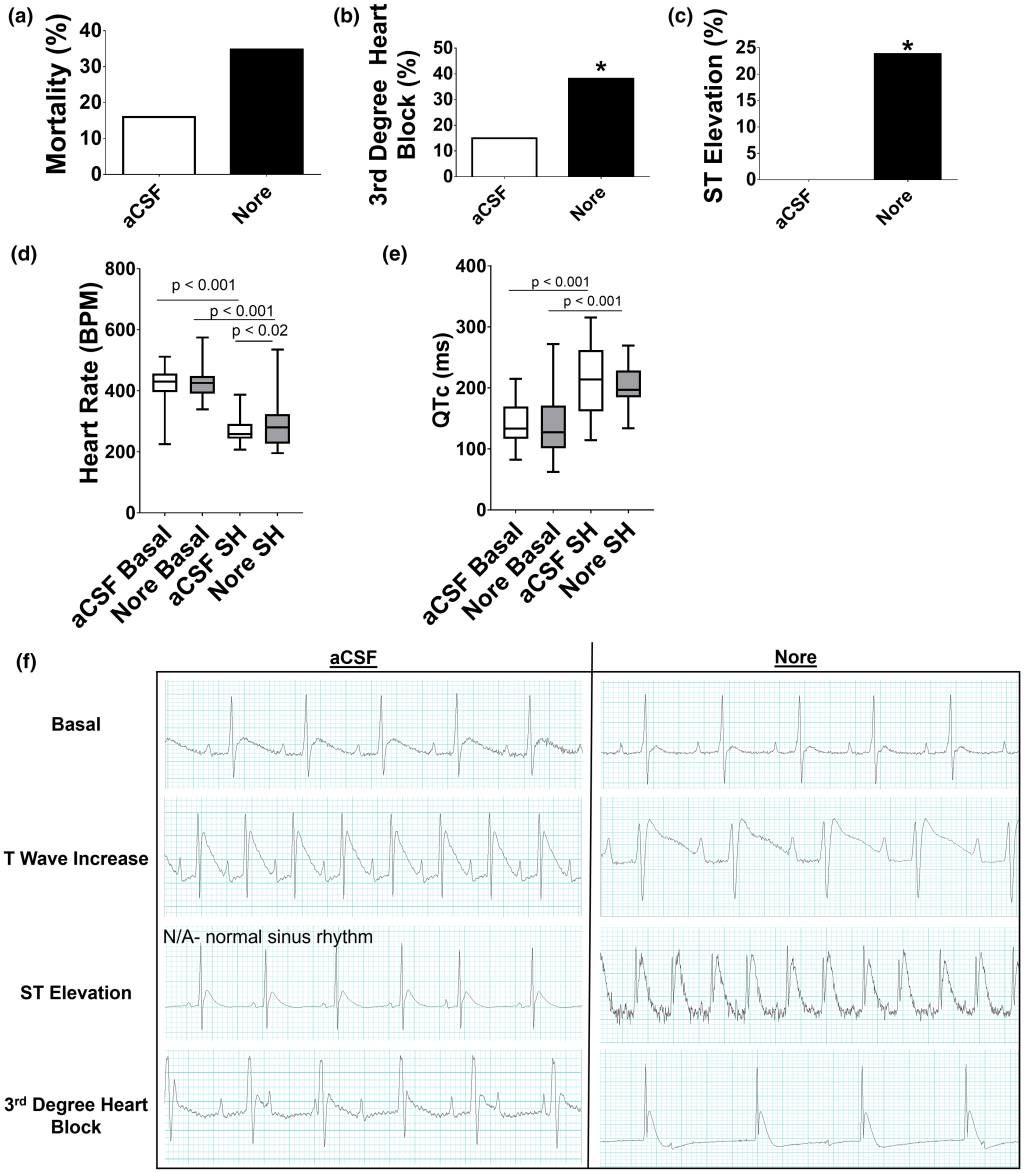
Experiment 1: Brain norepinephrine infusion ECG. (a) Mortality during severe hypoglycemia was 16% in aCSF (white bar) and 35% in Nore (black bar) but this was not statistically different. (b) Third‐degree heart block was increased in Nore compared to aCSF. (c) ST elevation was increased in Nore whereas aCSF did not experience ST elevation. (d) Heart rate decreased in both groups during severe hypoglycemia (SH) compared to their respective basals (*p* < 0.001). During severe hypoglycemia, Nore had a slight but significantly higher heart rate compared to aCSF (*p* < 0.02). (e) QTc prolongation occurred in both groups during severe hypoglycemia compared to basal with no differences between the two groups (*p* < 0.001). (f) Representative ECG from aCSF (left) and Nore (right). Basal: Both groups show normal sinus rhythm. T wave increase: Both groups had some rats that experienced this. ST elevation: This did not occur in aCSF. Instead normal sinus rhythm at similar timepoint during severe hypoglycemia is shown. Nore shows ST elevation. Third‐degree heart block: Rats from both groups experienced third‐degree heart block. Data are represented as box and whisker plots with median and interquaritle range and min to max values. *N* = 20–26/group.

Mortality during severe hypoglycemia was 16% in aCSF and 35% in Nore, but this did not reach significance (*p* < 0.19; Figure 2a). Third‐degree heart block (aCSF: 15%; Nore: 38%; *p* > 0.05) and ST elevation (aCSF: 0%; Nore: 24%; *p* < 0.019) were increased in Nore (Figure 2b,c).

Compared to basal, heart rate decreased during severe hypoglycemia in both groups (Figure 2d). Mean heart rate during severe hypoglycemia was increased in Nore (292 ± 81 BPM; *p* < 0.02) compared to aCSF (269 ± 39 BPM). QT prolongation occurred to a similar extent in both groups during severe hypoglycemia (Figure 2e). Figure 2f shows representative ECG graphs from both groups. Respiration and number of seizures during severe hypoglycemia were similar in both groups.

### 3.2 Experiment 2: Blockade of norepinephrine at nerve terminals

**FIGURE 3:**
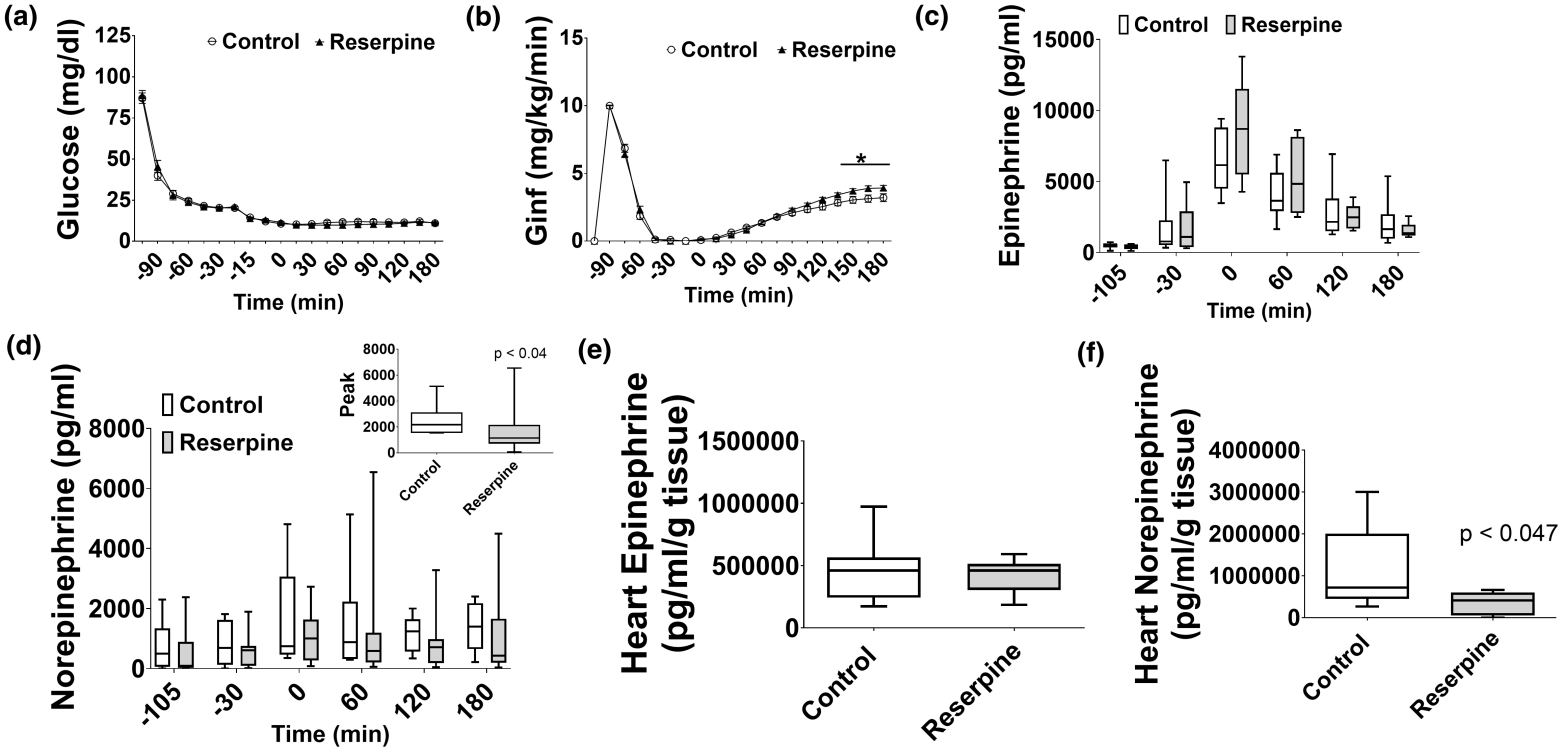
Experiment 2: Blockade of norepinephrine with reserpine clamp data. (a) Glucose levels were evenly matched between Control (white circles) and Reserpine (black triangles). (b) The glucose infusion rate required to maintain severe hypoglycemia was increased in Reserpine during the last 30 min compared to Control. (c) Plasma epinephrine was not different between the two groups. (d) Plasma norepinephrine was lower at all timepoints in Reserpine compared to Control. Insert: Peak norepinephrine was decreased in Reserpine (gray bar; *p* < 0.04) compared to control (white bar). (e) Heart epinephrine was not different between the two groups. (f) Heart norepinephrine was lower in Reserpine (gray bar; *p* < 0.047) compared to Control (white bar). Data are represented as box and whisker plots with median and interquaritle range and min to max values. *N* = 20–29/group.

During severe hypoglycemia, glucose levels were evenly matched between control (11.1 ± 0.88 mg/dL) and reserpine (11.1 ± 0.69 mg/dL; Figure 3a). Reserpine increased glucose infusion rates from 135 to 180 min of severe hypoglycemia (*p* < 0.05; Figure 3b). Cardiac norepinephrine and peak plasma norepinephrine during severe hypoglycemia were decreased by 72% and 53%, respectively, with reserpine compared to control (*p* < 0.05), whereas epinephrine was not different (Figure 3c–f).

**FIGURE 4:**
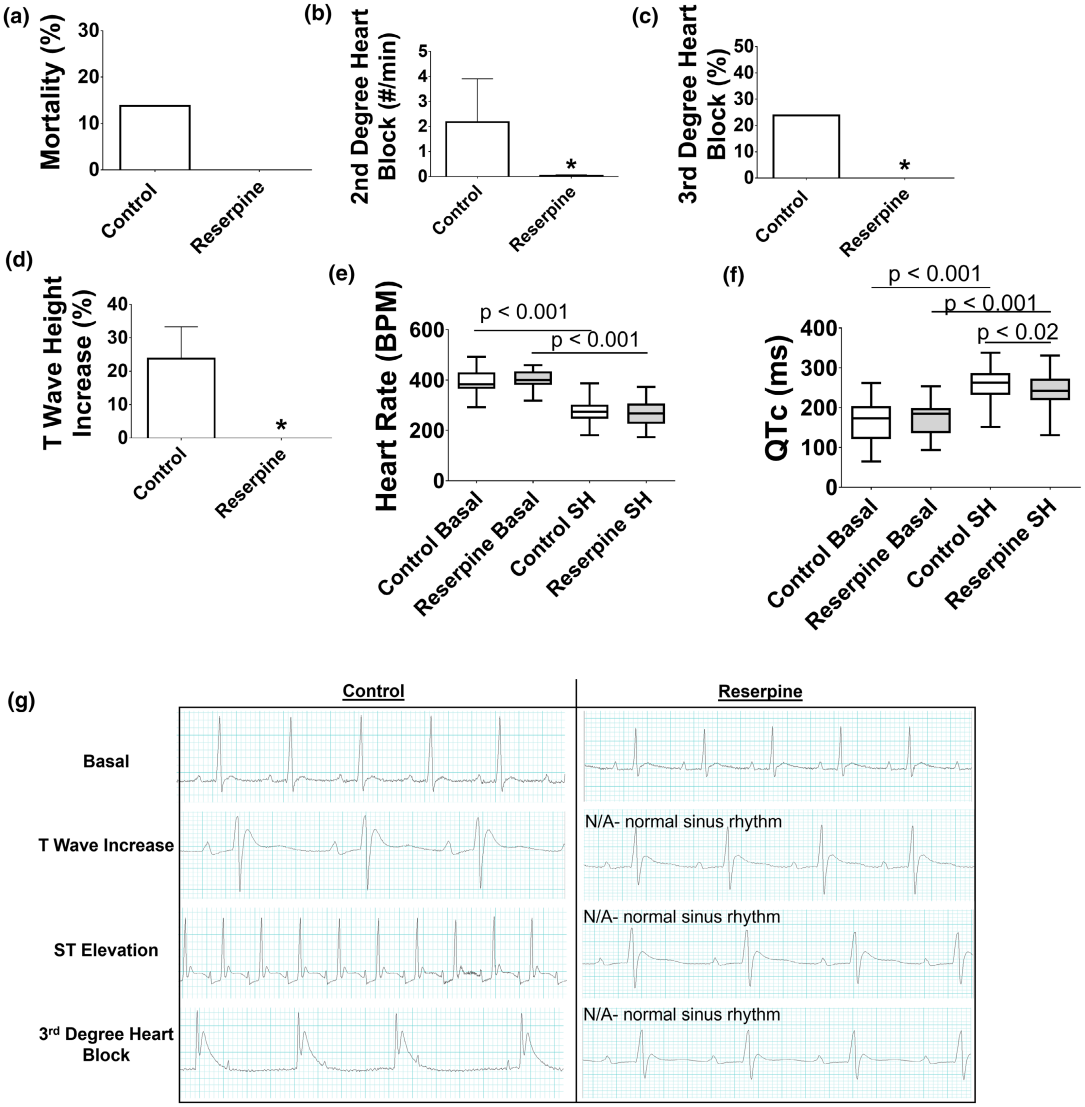
Experiment 2: Blockade of norepinephrine with reserpine ECG. (a) Mortality was completely prevented with Reserpine compared to 13.8% in Control (white bar) but this missed significance. (b) Second‐degree heart block was nearly prevented with Reserpine compared to Control. (c) Third‐degree heart block was prevented with Reserpine compared to 24% in Control. (d) T wave increase occurred in Control but was prevented with Reserpine. (e) Heart rate decreased during severe hypoglycemia compared to basal (*p* < 0.001) but to a similar extent in both groups. (f) QTc prolongation occurred in both groups compared to basal (*p* < 0.001) but to a lesser extent in Reserpine (*p* < 0.02). (g) Representative ECG from Control (left) and Reserpine (right) groups. Basal: Rats from both groups were in normal sinus rhythm. Shown under the control group is T wave increase, ST elevation, and third degree heart block. The rats in the Reserpine group did not have T wave increase, ST elevation, or third Degree heart block. Instead, an ECG at a similar timepoint during severe hypoglycemia (i.e., 1, 2, and 3 h) are shown with the rats in normal sinus rhythm. Data are represented as box and whisker plots with median and interquaritle range and min to max values. *N* = 20–29/group.

Although severe hypoglycemia‐induced mortality was completely prevented in rats with reserpine compared to 13.8% in controls, this did not reach significance (*p* < 0.13; Figure 4a). Second‐degree heart block was reduced 16.8‐fold in reserpine compared to controls (*p* < 0.05; Figure 4b). Third‐degree heart block was completely prevented in reserpine compared to an incidence of 24% in control rats (*p* < 0.05; Figure 4c). Additionally, T‐wave increase, an early marker of myocardial infarction, was prevented with reserpine compared to a 24% incidence in controls (*p* < 0.036; Figure 4d). ST elevation occurred in 19% of the controls while none of the reserpine rats had it, but this did not reach significance (*p* < 0.06).

Heart rate was reduced during severe hypoglycemia compared to basal in both groups (Figure 4e). Mean heart rate during severe hypoglycemia was not different in reserpine (265 ± 55 BPM) compared to control (278 ± 47 BPM) rats. QT prolongation occurred during severe hypoglycemia in both groups, but to a lesser extent in reserpine (243 ± 37 ms; *p* < 0.02) compared to control (258 ± 43 ms; Figure 4f). Figure 4g shows representative ECG graphs from both groups. There were no differences between the groups in respiration or seizure amount during severe hypoglycemia.

## 4 DISCUSSION

In order to reduce the incidence of death due to severe hypoglycemia in people with type 1 diabetes, a better understanding of the pathophysiology of severe hypoglycemia‐induced fatal cardiac arrhythmias is necessary. In this study, two experiments were performed in rats to test the hypothesis that norepinephrine increases fatal cardiac arrhythmias during severe hypoglycemia. The main findings were that during severe hypoglycemia, (1) brain norepinephrine infusion increased some types of cardiac arrhythmias, and (2) peripheral blockade of norepinephrine release from nerve terminals prevented major fatal cardiac arrhythmias.

It has been hypothesized that sympathetic activation is proarrhythmic. We found that brain norepinephrine infusion increased third‐degree heart block during severe hypoglycemia. Interestingly, ST elevation, a marker of myocardial infarction, was also increased by brain norepinephrine infusion compared to aCSF. In this study, 45% of the rats that died in both groups had ST elevation, whereas 100% of the rats that died experienced third‐degree heart block. While this is our first time reporting markers of myocardial infarction during severe hypoglycemia in this rat model, the prior studies may have overlooked these. It may be that a combination of third degree heart block and ST elevation increase risk of mortality during severe hypoglycemia.

With the dose of norepinephrine given, there was a significant rise in plasma norepinephrine and heart rate compared to aCSF rats indicating the dose was effective. Moreover, we performed preliminary experiments whereby brain norepinephrine was infused in the absence of hypoglycemia. A rise in blood glucose was witnessed during that study indicating the dose was effective.

The hypothesis that norepinephrine blockade could reduce arrhythmias during severe hypoglycemia was supported with the use of reserpine to block the release of norepinephrine, which prevented both second‐ and third‐degree heart block. Reserpine also eliminated T‐wave increase and ST elevation, both markers of myocardial infarction. Like our previous studies (Reno et al., 2019), arrhythmias could be prevented even in the presence of increased plasma epinephrine. This indicates that norepinephrine, and not epinephrine as previously thought, is the main regulator of severe hypoglycemia‐induced arrhythmias.

Reserpine is a competitive inhibitor of the vesicular monoamine uptake transporter (VMAT) that is responsible for the transport of cytosolic monoamines, such as norepinephrine, into synaptic vesicles in monoaminergic neurons (Roth & Stone, 1968). Reserpine blocks the transport of norepinephrine into the presynaptic vesicles reducing the concentration in the presynaptic terminals (Henry et al., 1987). Many of these postganglionic sympathetic neurons target the heart, and when activated, release norepinephrine, which binds to beta‐adrenergic receptors, increasing heart rate, blood pressure, and cardiac output (Hussain et al., 2024).

Reserpine is able to cross the blood–brain barrier. Thus, although reduction in arrhythmias with reserpine is thought to be primarily due to decreased norepinephrine in the heart, we cannot rule out that norepinephrine levels in the brain were also reduced, which could have influenced the results. Additionally, reserpine also irreversibly blocks the uptake of free intracellular dopamine, serotonin, and epinephrine into presynaptic vesicles (Carlsson et al., 1957). Altered dopamine and serotonin levels resulting from the use of reserpine may have factored into the differences in prevalence of arrhythmias as studies have shown dopamine and serotonin to be proarrhythmic (Argalious et al., 2005; Kaumann & Sanders, 1994; Patel et al., 2010).

Another limitation is that norepinephrine can synaptically regulate dopamine receptors within the ventral tegmental area (Liprando et al., 2004). A recent study showed that inhibition of the cardiac sympathetic nervous system in diabetic rats is regulated by dopamine (Rivera‐Mancilla et al., 2022). Thus, the increased brain norepinephrine in the current study could have caused an increase in dopamine, which could increase activation of the D_2/3/4_ receptors leading to inhibition of the cardiac sympathetic nervous system and reducing cardiac arrhythmias (Rivera‐Mancilla et al., 2022). This could be one reason some of the cardiac arrhythmias, like second‐degree heart block, were not increased by brain norepinephrine infusion. Future studies should look at the role of dopamine in severe hypoglycemia‐induced cardiac arrhythmias.

Finally, this study was conducted in only male rats. It is known that male and female effects on the cardiovascular system vary. Women have less effective baroreflex buffering of blood pressure as well as lower sympathoadrenal activity to support blood pressure (Hart et al., 2009). A relationship between total peripheral resistance, cardiac output, and muscle sympathetic nerve activity have been observed in males, but this relationship is not present in women (Hart et al., 2009). Furthermore, estrogen has been shown to attenuate vasoconstrictor responses and reduce total body norepinephrine spillover (Sudhir et al., 1997). Thus, future studies need to be conducted in female animals to fully understand how severe hypoglycemia might cause arrhythmias in this population.

Future studies in the clinical setting aimed to block norepinephrine in people at risk for severe hypoglycemia may prove effective at reducing cardiac abnormalities and arrhythmias.

## 5 CONCLUSION

In conclusion, norepinephrine is one of the main mediators of severe hypoglycemia‐induced fatal cardiac arrhythmias. Blocking the effects of norepinephrine with reserpine drastically reduces and prevents most types of fatal cardiac arrhythmias during severe hypoglycemia.

## AUTHOR CONTRIBUTIONS

E.H.N., M.E.C., and C.M.R.B. conducted the experiments, analyzed data, and wrote the manuscript. C.M.R.B. edited and revised the final manuscript.

## CONFLICT OF INTEREST STATEMENT

The authors disclose no conflict of interest.

## Data Availability

Data are available upon request.
